# Trends in Children’s Dietary Inflammatory Index and association with prediabetes in U.S. adolescents

**DOI:** 10.1038/s41387-024-00349-4

**Published:** 2024-11-18

**Authors:** Zisu Chen, Jing Wu, Kepeng Ai, Zhuying Bu, Wenquan Niu, Min Li

**Affiliations:** 1 Department of Pediatrics, Beijing Hospital of Traditional Chinese Medicine, Capital Medical University, Beijing, China; 2 Center for Evidence-Based Medicine, Capital Institute of Pediatrics, Beijing, China; 3 Department of Gastroenterology, Yan’an University Affiliated Hospital, Shaanxi, China

**Keywords:** Pre-diabetes, Nutrition

## Abstract

**Background and Objectives:**

Prediabetes is a high-risk state for diabetes. We aimed to illustrate secular trends in the Children’s Dietary Inflammation Index (C-DII) among U.S. adolescents and assess its association with prediabetes.

**Methods:**

Adolescents aged 12–18 years were collected from the National Health and Nutrition Examination Survey, 2001–2018. Prediabetes was defined based on Hemoglobin A1c, fasting glucose, and glucose tolerance levels. Risk was quantified by odds ratio (OR) and 95% confidence interval (CI).

**Results:**

A total of 13,684 adolescents were analyzed, representing a weighted total population of 33,351,181. C-DII scores declined significantly from 2001 to 2012 and increased from 2013 to 2018. The relationship between C-DII and prediabetes was roughly linear. When assigning the low C-DII scores as the reference, adolescents with medium and high C-DII scores were 1.22 (adjusted 95% CI: 1.04–1.44) and 1.25 (0.99–1.60) times more likely to have prediabetes. In subgroup analyses, the risk for prediabetes was significantly enhanced in boys (adjusted OR = 1.26 and 1.45 for medium and high C-DII scores, 95% CI: 1.05–1.51 and 1.09–1.92), and in adolescents living in poor families for medium (1.34 and 1.44, 1.08–1.67 and 1.07–1.95).

**Conclusions:**

Our findings indicate a V-shaped secular trend in C-DII scores from 2001 to 2018 in U.S. adolescents, with the nadir in 2011–2012, and the risk for prediabetes was significantly increased by over 20% in adolescents possessing medium or high C-DII scores.

## Introduction

Diabetes is the third most prevalent disorder in children [1]. The prevalence of diabetes was estimated to be 0.5% in children aged 12–19 years in 2015 [2]. Childhood diabetes can not only trigger morbid complications at earlier ages relative to diabetes developed in adulthood but also increase mortality risk by 30–200% relative to nondiabetic controls [1], underscoring the importance of early prevention. Prediabetes is a high-risk state for diabetes, and over 70% of prediabetic cases will progress to full diabetes [3]. Currently, approximately 1 in 5 adolescents is diagnosed with prediabetes [4], and primary prevention through close monitoring and active intervention of prediabetes before the onset of childhood diabetes should receive top public-health priority.

Chronic low-grade inflammation is widely believed to be implicated in the pathogenesis of diabetes. Some studies have shown that diverse dietary patterns and components have potential pro- or anti-inflammatory properties, and they can affect disease risk by multiple mechanisms of action, including intestinal microbiota, oxidative stress, and energy balance [5]. To assess the overall dietary quality of inflammation in adults, a composite index, Dietary Inflammatory Index (DII), has been coined and it has advantages over individual dietary compounds by considering complex interactions of multiple nutrients and compounds within foods and dietary patterns [5, 6]. In particular, children’s DII (C-DII) is suitable for children. There is evidence that DII was significantly associated with prediabetes in adults [7]; however, this association remains unexplored in adolescents.

Additionally, it is widely recognized that monitoring secular trends in DII scores plays an indispensable role in public-health surveillance, and it can facilitate the identification, implementation, and evaluation of early prevention strategies targeting chronic diseases connected with diet-based inflammation. The secular trends in DII scores have been outlined in adults [8], while no data are available in adolescents.

To fill the above research gaps and yield more information, we aimed to illustrate secular trends in C-DII among U.S. adolescents first, and then to assess its association with prediabetes risk by analyzing data from the National Health and Nutrition Examination Survey (NHANES), 2001–2018.

## Methods

### Data source and study participants

NHANES is a research program designed to assess the health and nutritional status of adults and children in the U.S. by uniquely combining interviews and physical examinations. All survey protocols were approved by the research ethics review board at the National Center for Health Statistics, and written informed consent was obtained from all respondents prior to participation.

Participants from the NHANES, 2001–2018, totaling 9 survey cycles, were enrolled. Eligible participants should satisfy the following criteria concurrently: (i) adolescents aged between 12 and 19 years old; (2) participants with available first-day dietary interview data. In total, 13,684 adolescents were retained in the final analysis.

### C-DII assessment

C-DII was formulated in a way like DII. Differing from 45 food parameters used in adulthood DII, C-DII simply used 25 of them due to dietary differences between adults and children. The calculation of C-DII rested on 24-h recall dietary nutrient intake, including carbohydrate, protein, fat, alcohol, fiber, cholesterol, saturated fat, monounsaturated fatty acids, polyunsaturated fatty acids, niacin, thiamin, riboflavin, vitamin B12, vitamin B6, iron, magnesium, zinc, selenium, vitamin A, vitamin C, vitamin E, folic acid, and beta carotene. Based on the association with six inflammatory biomarkers (interleukin-1beta, interleukin-4, interleukin-6, interleukin-10, tumor necrosis factor-alpha, and C-reactive protein), each food is assigned a specific nutrient DII score. The validity of C-DII has been basically confirmed, i.e., it is positively associated with markers of chronic inflammation and oxidative stress, and this association is the same whether in adolescents or children [9, 10].

In detail, C-DII was calculated in three steps. Firstly, the difference between individual’s and global’s daily mean intake divided by standard deviation equals a Z score. Secondly, to avoid “right skewing” and achieve the symmetrical distribution, the Z score is converted to a percentile value and doubled before subtracting “1”. Thirdly, the obtained value times each nutrient’s dietary inflammation index score is the DII of each food nutrient. Overall C-DII scores are the sum of each score obtained by previous steps [9, 10].

In this study, eligible adolescents were evenly divided into 3 groups according to C-DII scores: low C-DII group (−4.91 ≤ C-DII ≤ 0.75), medium C-DII group (0.75 < C-DII ≤ 2.33), and high C-DII group (2.33 < C-DII ≤ 4.97) [11].

### Prediabetes definition

Prediabetes was defined according to the following criteria: (i) Hemoglobin A1c (HbA1c) levels: 5.7%–6.4%; (ii) fasting glucose levels 100–125 mg/dl; (iii) glucose tolerance levels: 140–199 mg/dl [7, 12]. It can be defined by meeting one of these criteria.

### Covariates

Age, sex, race/ethnicity, poverty income ratio (PIR), and serum cotinine were included. All covariates under study can be accessed/downloaded from the official NHANES website (https://www.cdc.gov/nchs/nhanes/index.htm).

### Statistical analyses

All analyses in this study have incorporated oversampling, clustering, and stratification procedures to estimate the representative statistics of the general U.S. adolescent population given the multistage complex design of NHANES. Continuous variables are represented as weighted mean (95% confidence interval [CI]). Categorical variables are represented as weighted proportions. Wilcoxon rank-sum test for skewed continuous variables and *χ*^2^ test for categorical variables were used for group-based comparisons. Overall and subsidiary weighted mean C-DII scores in U.D. adolescents across survey cycles were determined. The nonlinear relationship of C-DII with prediabetes was examined by restricted cubic spline (RCS) analysis. Weighted logistic regression analysis was implemented to assess the association between C-DII and prediabetes, with and without controlling age, sex, race, PIR, serum cotinine.

Two-sided P less than 0.05 was considered statistically significant. Data were analyzed using Stata software version 17 (StataCorp LP, TX, USA) and R programming environment version 4.2.3.

## Results

### Baseline characteristics

Weighted baseline characteristics of study adolescents are presented in Table 1. A total of 13,684 adolescents were included in this study, representing a weighted total population of 33,351,181. Weighted mean age was 15.5 years and 50.6% of adolescents were boys. The percentage of prediabetes was 20.1%.

**Table 1:** Demographic characteristics in US adolescents by prediabetes from NHANES, 2001–2018^a^.

Characteristics	Total	Without prediabetes	With prediabetes	*P*-value
Unweighted sample size	13,684	10,908	2748	
Weighted sample size	33,351,181	27,168,878	6,108,579	
Age, years, mean (95% CI)	15.49 (15.42, 15.56)	15.51 (15.43, 15.59)	15.40 (15.27, 15.54)	<0.001
Sex, %				
Boys	50.64	49.41	56.04	<0.001
Girls	49.36	50.59	43.96	
Race/ethnicity, %				
Hispanic	19.90	18.94	24.23	<0.001
Non-Hispanic White	57.82	59.82	48.84	
Non-Hispanic Black	14.52	13.52	18.95	
Other race	7.76	7.72	7.98	
Poverty income ratio, mean (95% CI)	2.50 (2.41, 2.59)	2.55 (2.46, 2.65)	2.24 (2.13, 2.36)	<0.001
Cotinine, ng/mL, mean (95% CI)	17.70 (15.43, 19.97)	17.53 (15.01, 20.05)	18.28 (14.02, 22.55)	0.420
Glucose, mmol/L, mean (95% CI)	94.80 (94.21, 95.39)	91.29 (90.57, 92.00)	100.26 (99.40, 101.11)	<0.001
OGTT, mg/dl, mean (95% CI)	97.61 (96.14, 99.08)	92.35 (90.62, 94.08)	105.08 (103.04, 107.12)	<0.001
HbA1c, %, mean (95% CI)	5.21 (5.19, 5.22)	5.16 (5.15, 5.17)	5.37 (5.35, 5.39)	<0.001
DII, mean (95% CI)	1.47 (1.42, 1.53)	1.46 (1.41, 1.52)	1.52 (1.41, 1.62)	<0.001

*NHANES* National Health and Nutrition Examination Survey, *CI* confidence interval, *OGTT* oral glucose tolerance test, *DII* Dietary Inflammatory Index.

^a^Nationally representative estimates were derived by using survey weights.

### C-DII secular trends

Table 2 shows the secular trends in weighted mean C-DII scores across NHANES cycles. Obviously, C-DII scores declined significantly from 2001 to 2012 as reflected by the Mann–Kendall trend test, and then increased from 2013 to 2018.

**Table 2:** Distribution of C-DII scores in US adolescents from NHANES 10 cycles^a^.

Characteristics	2001–2002 (*n* = 9701)	2003–2004 (*n* = 8894)	2005–2006 (*n* = 9169)	2007–2008 (*n* = 9118)	2009–2010 (*n* = 9623)	2011–2012 (*n* = 8389)	2013–2014 (*n* = 8531)	2015–2016 (*n* = 8327)	2017–2018 (*n* = 7484)
**Overall sample**	1.64 (1.52, 1.75)	1.58 (1.42, 1.75)	1.59 (1.47, 1.71)	1.49 (1.32, 1.65)	1.41 (1.25, 1.57)	1.31 (1.14, 1.47)	1.35 (1.21, 1.50)	1.42 (1.29, 1.55)	1.49 (1.31, 1.68)
**Age, years**									
12–15	1.75 (1.60, 1.91)	1.66 (1.47, 1.86)	1.61 (1.43, 1.79)	1.60 (1.36, 1.84)	1.40 (1.20, 1.61)	1.47 (1.23, 1.71)	1.49 (1.34, 1.64)	1.36 (1.14, 1.59)	1.48 (1.29, 1.66)
16–19	1.51 (1.35, 1.66)	1.50 (1.29, 1.72)	1.56 (1.42, 1.71)	1.38 (1.18, 1.58)	1.42 (1.25, 1.59)	1.15 (0.97, 1.33)	1.24 (1.00, 1.47)	1.49 (1.33, 1.64)	1.51 (1.24, 1.77)
**Sex**									
Boys	1.28 (1.09, 1.47)	1.33 (1.14, 1.52)	1.30 (1.15, 1.45)	1.24 (0.99, 1.49)	1.04 (0.77, 1.31)	0.78 (0.62, 0.93)	1.05 (0.93, 1.18)	1.19 (1.02, 1.37)	1. 27 (1.03, 1.52)
Girls	1.99 (1.84, 2.14)	1.86 (1.70, 2.02)	1.90 (1.73, 2.08)	1.74 (1.49, 1.98)	1.75 (1.62, 1.88)	1.85 (1.59, 2.11)	1.66 (1.40, 1.92)	1.65 (1.51, 1.79)	1.73 (1.55, 1.90)
**Race/ethnicity**									
Hispanic	1.48 (1.34, 1.62)	1.24 (1.01, 1.48)	1.46 (1.35, 1.58)	1.53 (1.26, 1.80)	1.25 (1.10, 1.41)	0.97 (0.71, 1.23)	1.21 (0.92, 1.49)	1.42 (1.30, 1.54)	1.42 (1.21, 1.63)
Non-Hispanic White	1.63 (1.47, 1.79)	1.63 (1.42, 1.84)	1.55 (1.35, 1.74)	1.38 (1.14, 1.62)	1.46 (1.22, 1.69)	1.36 (1.09, 1.63)	1.41 (1.22, 1.61)	1.44 (1.23, 1.64)	1.55 (1.20, 1.89)
Non-Hispanic Black	1.93 (1.73, 2.14)	1.66 (1.40, 1.92)	1.80 (1.61, 1.99)	1.83 (1.55, 2.11)	1.59 (1.33, 1.85)	1.66 (1.46, 1.85)	1.50 (1.32, 1.69)	1.63 (1.36, 1.90)	1.68 (1.43, 1.93)
Other race	1.51 (0.92, 2.10)	1.79 (1.31, 2.26)	1.74 (1.49, 1.99)	1.62 (1.17, 2.07)	1.09 (0.70, 1.47)	1.20 (0.84, 1.55)	1.13 (0.49, 1.76)	1.03 (0.74, 1.32)	1.20 (0.99, 1.41)
**Poverty income ratio, %**									
0–1.3	1.84 (1.64, 2.04)	1.58 (1.28, 1.88)	1.61 (1.43, 1.78)	1.57 (1.20, 1.93)	1.43 (1.23, 1.64)	1.33 (1.17, 1.49)	1.46 (1.19, 1.74)	1.61 (1.44, 1.79)	1.49 (1.15, 1.83)
1.3–3.5	1.68 (1.54, 1.82)	1.66 (1.50, 1.83)	1.70 (1.53, 1.87)	1.71 (1.52, 1.91)	1.55 (1.30, 1.79)	1.42 (1.17, 1.68)	1.36 (1.08, 1.63)	1.46 (1.28, 1.65)	1.53 (1.33, 1.72)
≥3.5	1.36 (1.18, 1.53)	1.49 (1.27, 1.72)	1.44 (1.21, 1.67)	1.30 (1.13, 1.46)	1.32 (1.11, 1.53)	1.11 (0.76, 1.47)	1.17 (0.86, 1.48)	1.17 (0.94, 1.40)	1.39 (1.00, 1.78)
**Cotinine, ng/mL**									
No smoking	1.47 (1.26, 1.67)	1.21 (1.06, 1.38)	1.39 (1.18, 1.61)	1.40 (1.11, 1.69)	1.25 (1.10, 1.40)	1.16 (1.02, 1.30)	1.17 (1.00, 1.35)	1.20 (1.03, 1.36)	1.37 (1.15, 1.58)
Second-hand smoking	1.70 (1.55, 1.84)	1.78 (1.60, 1.97)	1.72 (1.53, 1.91)	1.53 (1.27, 1.79)	1.52 (1.26, 1.79)	1.42 (1.09, 1.75)	1.48 (1.25, 1.70)	1.70 (1.45, 1.95)	1.65 (1.46, 1.83)
Smoker	1.84 (1.63, 2.06)	1.70 (1.46, 1.94)	1.70 (1.56, 1.84)	1.55 (1.29, 1.81)	1.58 (1.34, 1.83)	1.49 (1.04, 1.93)	1.70 (1.44, 1.97)	1.64 (1.44, 1.83)	1.60 (1.21, 1.99)

*NHANES* National Health and Nutrition Examination Survey, *DII* Dietary Inflammatory Index. Data are expressed as mean (95% confidence interval).

^a^Nationally representative estimates were derived by using survey weights.

**Fig. 1:**
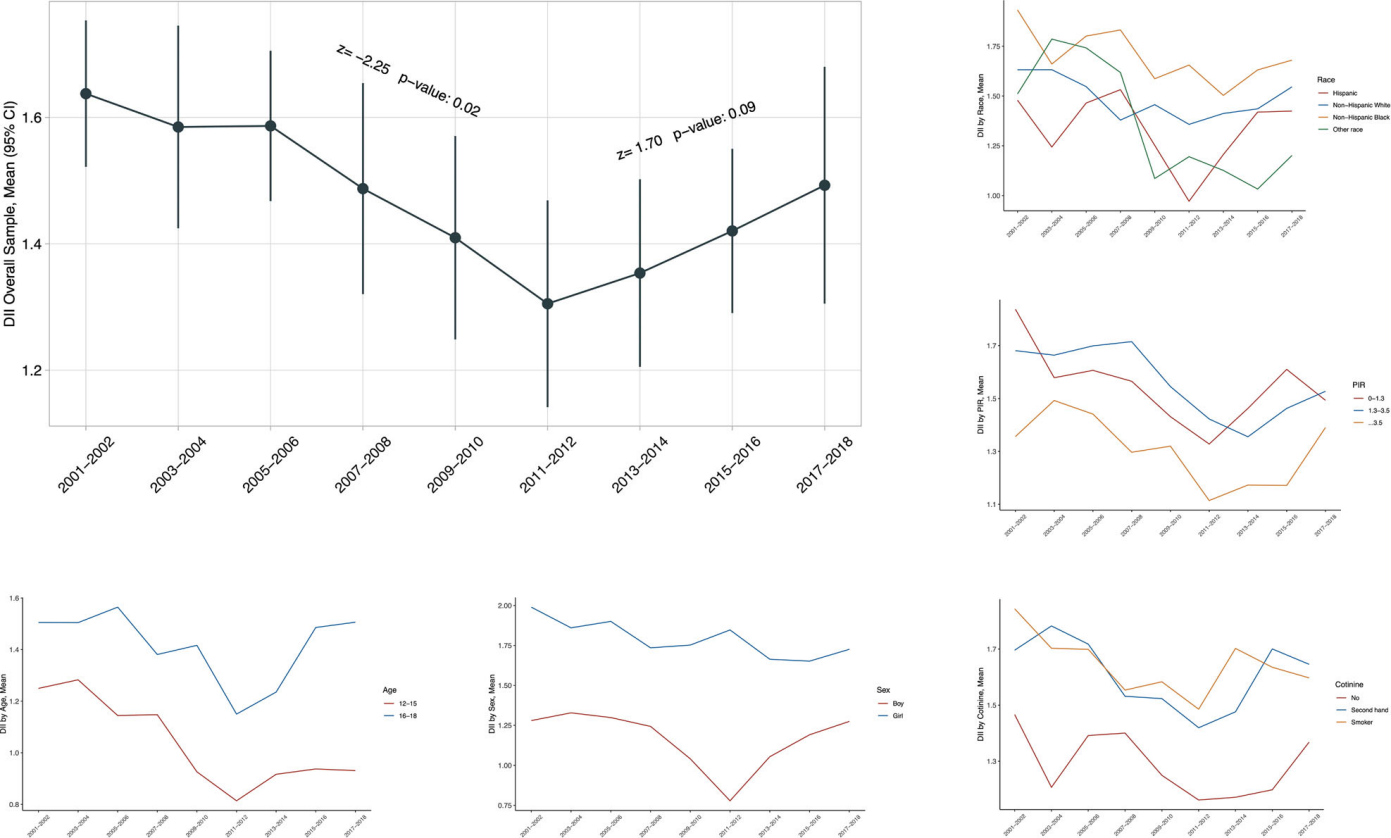
Weighted mean Dietary Inflammatory Index (DII) scores in US adolescents from NAHES, 2001–2018^*^. The Mann–Kendall test was used to assess the trends. ^*^Nationally representative estimates were derived by using survey weights.

Secular trends in C-DII scores varied across subpopulations stratified by age, sex, race/ethnicity, PIR, and serum cotinine, respectively (Fig. 1). Specifically, C-DII was higher among older adolescents (16–19 years) than younger adolescents (12–15 years), and among girls than boys. Although no significance was noted by race/ethnicity groups, non-Hispanic Blacks maintained higher C-DII scores than the others. Adolescents living in wealthy families (PIR > 1) had lower C-DII scores than poor families (PIR ≤ 1). Non-smoking adolescents had lower C-DII scores than smoking adolescents.

### C-DII and prediabetes: overall analyses

**Fig. 2:**
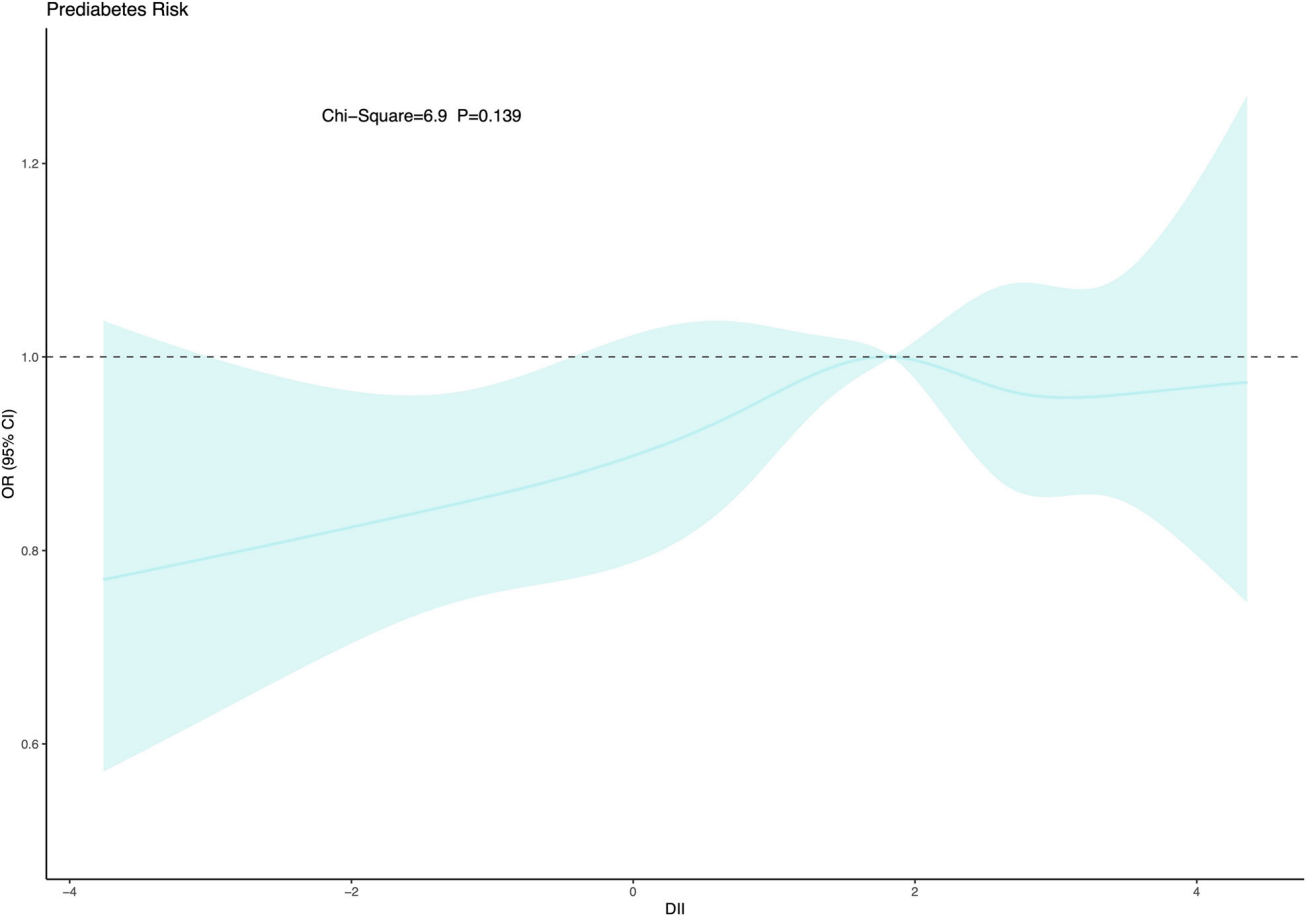
Dose-dependent assessment of Dietary Inflammatory Index (DII) with prediabetes in US adolescents.

The relationship between C-DII and prediabetes was roughly linear, as the nonlinear test was nonsignificant (Fig. 2).

**Fig. 3:**
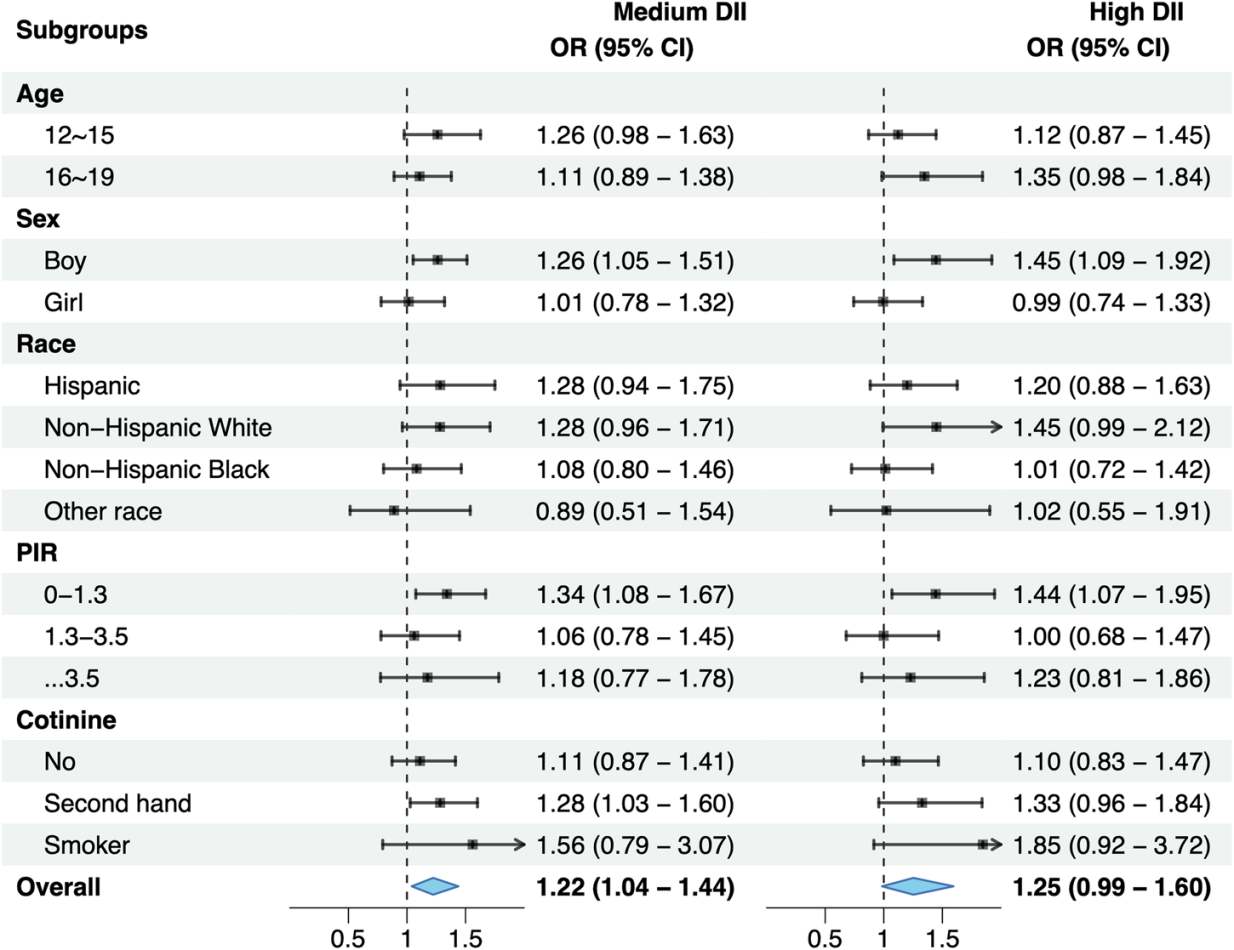
Overall and subgroup association of Dietary Inflammatory Index (DII) (high and medium versus low) in survey-weighted logistic regression analyses^*^. OR odds ratio, 95% CI 95% confidence interval, PIR poverty income ratio. Effect-size estimates were adjusted for age, sex, race and ethnicity, poverty income ratio, cotinine. ^*^Nationally representative estimates were derived by using survey weights.

The association of C-DII scores in tertiles with prediabetes is presented in Fig. 3. When assigning the low C-DII scores as the reference, adolescents with medium and high C-DII scores were 1.22 (95% CI: 1.04–1.44) and 1.25 (95% CI: 0.99–1.60) times more likely to have prediabetes after adjusting for confounding factors.

### C-DII and prediabetes: subsidiary analyses

The association between C-DII and prediabetes was also explored in subsidiary analyses by age, sex, race/ethnicity, PIR, and serum cotinine, respectively (Fig. 2). For the comparison of medium versus low C-DII scores, the risk for prediabetes was enhanced in adolescents aged 12–15 years (OR = 1.26, 95% CI: 0.98–1.63), and for the comparison of high versus low C-DII scores, enhanced risk was seen in adolescents aged 16–19 years (OR = 1.35, 95% CI: 0.98–1.84). By sex, both medium and high C-DII scores were associated with increased prediabetes risk only in boys (OR = 1.26 and 1.45, 95% CI: 1.05–1.51 and 1.09–1.92, respectively).

There was no hint of statistical significance when stratified by race/ethnicity. Regarding PIR, the association with prediabetes was significant in adolescents living in poor families for medium (OR = 1.34, 95% CI: 1.08–1.67) and high (OR = 1.44, 95% CI: 1.07–1.95) C-DII scores relative to low scores. By cotinine exposure, there was marginal significance for second-hand cotinine exposure for medium C-DII scores (OR = 1.28, 95% CI: 1.03–1.60).

## Discussion

The aims of this study were two-fold: to illustrate secular trends in C-DII among U.S. adolescents and to assess the association of C-DII with prediabetes. It is worth noting a V-shaped secular trend in C-DII scores from 2001 to 2018 in U.S. adolescents, with the nadir in 2011–2012. Moreover, the risk for prediabetes was linearly increased with increasing C-DII scores and was significantly increased by over 20% in adolescents possessing medium or high scores, especially with male sex, living in poor families, and exposed to second-hand cotinine. To our knowledge, this is thus far the first study that has explored the association between C-DII and prediabetes in adolescents in the literature.

Unlike the secular trends in DII among U.S. adults [8], which increased from 1999 to 2004, declined until 2009 and then increased slightly from 2009 to 2014, we among U.S. adolescents found an obvious declining trend in C-DII scores and then an increasing trend afterward, following a V-shaped pattern. Such differences in trends between adults and adolescents are not surprising, in view of differing age intervals and varying food components. A note of caution ought to be sounded as C-DII scores are continuously increased at the end of the NHANES survey and are on average greater than the upper threshold, meaning that U.S. adolescents are currently consuming more pro-inflammatory diets. What’s more, high C-DII scores are closely linked to chronic inflammation, the underlying cause of cardiometabolic disease and cancer [13–15].

It is intriguing to wonder why this dietary metric of U.S. adolescents had shown a V-shaped change over the past decades. Regarding potential explanations for this, it is reasonable to posit that C-DII secular trends are associated with diabetes-related trends, as both diabetes and C-DII are related to dietary behavior. The C-DII trends observed herein partially align with diabetes trends among American adolescents. The prevalence of diabetes in Korea has increased sharply between 2007–2009 and 2016–2018, while the increasing tend to slow down during 2010–2012 [16]. This stagnation of growth can also be observed in adults [2, 17]. The exact reason for this phenomenon is elusive. In general, the trend of increasing obesity closely parallels that of increasing diabetes.

It is well established that chronic inflammation plays a critical important role in the pathogenesis of diabetes and prediabetes [18–20]. As a dietary index of chronic inflammation, DII is proposed to be a powerful harbinger in predicting the risk of diabetes [21, 22], while the evidence linking DII to prediabetes is sparse and inconsistent in the medical literature. Lee and colleagues found that the prevalence of prediabetes defined by HbA1c abnormalities has significantly increased over time among U.S. adolescents [23]. Some studies have shown that DII was significantly associated with prediabetes, whereas others failed to support this claim. For instance, in 7926 adults from NHANES, the highest tertile of DII scores was associated with over 40% increased risk of prediabetes compared with those in the first tertile [7]. Another study of 20,762 general U.S. adults with different glycemic statuses demonstrated a positive correlation between high DII scores and the prevalence of prediabetes [24]. By contrast, in a cohort of primarily Hispanic young adults, no hint of significance was seen for the association between DII scores and prediabetes [25]. In 2975 adults from Iran, there was no significant association between DII and risk of type 2 diabetes and insulin resistance [26]. Although the reasons behind the above lines of inconsistent findings are not fully understood, we speculate that the diverse origins of the study population, different eligibility criteria, and various statistical powers mattered.

As an extension of previous studies and bearing the possible reasons mentioned above, we attempted to assess the association of C-DII, the children’s version of DII, with prediabetes among 13,684 U.S. adolescents 12–18 years of age. To eliminate confounding impact, a large panel of baseline factors was controlled from statistical aspects and stratified in subgroup explorations. Noteworthily, high C-DII was significantly and independently associated with the increased risk of prediabetes, in line with the recent results in the study by Shu and colleagues [7]. There are two possible mechanisms underlying the association between DII and prediabetes. One is that there is a positive association between DII scores and individual biomarkers of low-grade inflammation [27]. Diet may act on chronic diseases such as prediabetes and diabetes by regulating low-grade inflammation [28]. Another possible mechanism is that high DII scores can impact prediabetes and diabetes by promoting the development of obesity [7, 29, 30]. Based on the above evidence, proper intervention of daily diets could form an effective preventive strategy for the onset and progression of prediabetes in adolescents.

There are several limitations in our study. Firstly, self-reported diets cannot rule out recall errors. To overcome this, 24 h recall dietary data were extracted. Secondly, this study was cross-sectional in design, which cannot infer the casual relationship between C-DII scores and prediabetes. Thirdly, although multi-covariates were under control, unaccounted residual confounding cannot be fully excluded. Although our findings indicate significance between C-DII and diabetes, the detected effects are weak in the large sample.

Despite these limitations, our findings indicate that there was a V-shaped secular trend in C-DII scores from 2001 to 2018 in U.S. adolescents, with the nadir in 2011–2012, and that the risk for prediabetes was significantly increased by over 20% in adolescents possessing medium or high scores. Echoing from our findings, monitoring self-dietary patterns may represent a good alternative option for the prevention of prediabetes, and it is expected that doing so can reduce diabetes healthcare costs and prevent diabetes by improving the quality of life for youth in the long term.
