# Correction: Trends in Children’s Dietary Inflammatory Index and association with prediabetes in U.S. adolescents

**DOI:** 10.1038/s41387-025-00393-8

**Published:** 2025-10-08

**Authors:** Zisu Chen, Jing Wu, Kepeng Ai, Zhuying Bu, Wenquan Niu, Min Li

**Affiliations:** 1 Department of Pediatrics, Beijing Hospital of Traditional Chinese Medicine, Capital Medical University, Beijing, China; 2 Center for Evidence-Based Medicine, Capital Institute of Pediatrics, Beijing, China; 3 Department of Gastroenterology, Yan’an University Affiliated Hospital, Shaanxi, China

**Keywords:** Pre-diabetes, Nutrition

Correction to: *Nutrition and Diabetes* 10.1038/s41387-024-00349-4, published online 18 November 2024

In this article, Min Li, Department of Pediatrics, Beijing Hospital of Traditional Chinese Medicine, Capital Medical University, Beijing, China was incorrectly denoted as the corresponding author, but it should have been Wenquan Niu, Center for Evidence-Based Medicine, Capital Institute of Pediatrics, Beijing, China.

The original article has been corrected.

